# Machine Learning Algorithms to Predict In-Hospital Mortality in Patients with Traumatic Brain Injury

**DOI:** 10.3390/jpm11111144

**Published:** 2021-11-04

**Authors:** Sheng-Der Hsu, En Chao, Sy-Jou Chen, Dueng-Yuan Hueng, Hsiang-Yun Lan, Hui-Hsun Chiang

**Affiliations:** 1 Division of Traumatology, Department of Surgery, Tri-Service General Hospital, National Defense Medical Center, Taipei 10490, Taiwan; f1233j@yahoo.com.tw; 2 Department of Medical Affairs, Song Shan Branch, Tri-Service General Hospital, Taipei 10490, Taiwan; johnny2cindy@gmail.com; 3 Department of Emergency Medicine, Tri-Service General Hospital, National Defense Medical Center, Taipei 10490, Taiwan; syjou.chen@gmail.com; 4 Department of Neurological Surgery, Tri-Service General Hospital, National Defense Medical Center, Taipei 10490, Taiwan; hondy2195@yahoo.com.tw; 5 School of Nursing, National Defense Medical Center, No 161, Section 6, Minquan E. Road, Neihu District, Taipei 10490, Taiwan; shinnylan@gmail.com

**Keywords:** electronic medical record, machine learning, Glasgow coma scale (GCS), injury severity scale (ISS), blood pressure, traumatic brain injury (TBI), in-hospital mortality

## Abstract

Traumatic brain injury (TBI) can lead to severe adverse clinical outcomes, including death and disability. Early detection of in-hospital mortality in high-risk populations may enable early treatment and potentially reduce mortality using machine learning. However, there is limited information on in-hospital mortality prediction models for TBI patients admitted to emergency departments. The aim of this study was to create a model that successfully predicts, from clinical measures and demographics, in-hospital mortality in a sample of TBI patients admitted to the emergency department. Of the 4881 TBI patients who were screened at the emergency department at a high-level first aid duty hospital in northern Taiwan, 3331 were assigned in triage to Level I or Level II using the Taiwan Triage and Acuity Scale from January 2008 to June 2018. The most significant predictors of in-hospital mortality in TBI patients were the scores on the Glasgow coma scale, the injury severity scale, and systolic blood pressure in the emergency department admission. This study demonstrated the effective cutoff values for clinical measures when using machine learning to predict in-hospital mortality of patients with TBI. The prediction model has the potential to further accelerate the development of innovative care-delivery protocols for high-risk patients.

## 1. Introduction

Traumatic brain injury (TBI) creates an enormous health and economic burden for the visitors to an emergency department (ED) [1]. In addition to the high medical expenses, TBI patients are confronted with physical-psychological threats associated with suffering, hospitalization, disability, and death [2,3]. The essence of emergency nursing per se is high mobility and agility and dependence on rapid assessment, decision making, and emergency management [4]. Treatment-limiting clinical decisions are often required during the acute phase immediately after TBI. The very process of decision making is characterized by both a lack of objective criteria and absence of a validated prognostic model that can predict the relevant outcome [5]. It is crucial for patients, proxies, and medical professionals to make shared treatment decisions based on accurate long-term predictions from a validated prognostic model informed by clinical measures at ED admission following the acute phase of TBI.

### 1.1. Background

Scores on the Glasgow coma scale (GCS) are an important indicator of severity of TBI [4]. Likewise, the GCS is the most commonly used measure of one’s level of consciousness following a TBI [6]. The GCS is composed of three subscales that independently measure eye opening, verbal response, and motor response. It has been used to predict mortality following TBI [7]. Previous study indicates that when given at arrival to the hospital, GCS is the most significant predictor of overall mortality among TBI patients [8]. GCS ≤ 8 is the cutoff value for defining a TBI as severe [9]. However, the success of GCS scores obtained at the time of the trauma in predicting mortality has not been supported by other studies when the scores were obtained in the triage room [10]. A single GCS score may not be a reliable indicator of mortality, but GCS scores obtained both on arrival and prior to arrival have been found to be highly correlated with mortality and symptom severity in TBI patients [8].

The injury severity scale (ISS) is the best predictor of mortality in trauma patients [11]. The abbreviated injury scale (AIS) has been recognized since its development in 1971 as the gold standard for obtaining injury severity ratings [12]. The AIS assigns a severity score to an injury on a scale from one (minor) to six (major) at each of the following locations: head, face, neck, thorax, abdomen, spine, upper and lower extremities, and external/other [13]. Although AIS is commonly used to classify severity, AIS and GCS are more highly correlated in cases of severe brain injury than in cases of mild or moderate injury [13]. The AIS score cannot be calculated by hand at the scene of the trauma as it requires a manual review of the patient’s record. The ISS score is calculated by taking the highest AIS score for each body region, squaring it, and then summing the three highest scores [14]. In the literature, ISS scores are categorized as <9, 9–15, 16–24, and ≥25 [13]. A study focusing on prognostic factors for TBI mortality in Japan likewise found that the ISS was a significant predictor of TBI mortality in patients [15]. Positive correlations have also been consistently found between the ISS and TBI mortality in Korea, Europe, and America [13,16,17]. Another cross-sectional retrospective study revealed that the GCS and ISS both have the capacity to predict mortality in young children with TBI, with the cutoff values for the ISS and GCS being 15 and 11, respectively [11]. Although ISS criteria alone may not predict short-term outcomes, prognostic models that also include GCS scores play an important role in predicting mortality in the hospital in TBI patients [10].

Injury-induced change in initial physiological measures such as systolic blood pressure (SBP), diastolic blood pressure (DBP), heart rate (HR) and pulse pressure (PP) variability can provide useful prognostic information about TBI patients [18,19,20]. SBP on arrival at the ED is correlated with mortality in patients with major TBI [21]. Previous studies found that the depth and duration of out-hospital hypotension were associated with increased TBI mortality [22]. Other studies found that hypotension and hypertension in the ED seemed to be associated with increased mortality in TBI patients [20], and the mortality rate was higher when SBP was <120 mm Hg or ≥140 mm Hg in the ED [23]. It was found that the hypotension threshold required a higher SBP (<110 mm Hg) in isolated moderate to severe TBI patients [24]. Moreover, DBP was an early predictor of mortality in major trauma patients across all stages of care [25]. However, no one has been able to identify a unique physiological cutoff point that predicts mortality in patients with TBI [21]. Clinicians should not rely on blood pressure criteria alone to diagnose TBI. Even SBP alone may not be a reliable indicator of TBI mortality; hemodynamic parameters such as HR may need to be added to the vital signs criteria to make a rigorous prediction of TBI patients’ consciousness level after traumatic injury [15]. A previous study suggested that SBP lower than HR can indicate a poor prognosis for trauma patients [9]. Mean PP dichotomized at 72.17 mm Hg provided a cutoff point for mortality in brain trauma patients [26]. Above all, physiomarkers such as BP, HR and PP are significant predictors of in-hospital mortality in TBI patients.

Not only clinical outcomes following TBI, but also demographics, have been shown to be significant prognostic factors for TBI patients [27]. A previous study revealed that TBI mortality increased substantially with age [28]. A retrospective analysis of data from over 1.7 million people hospitalized from 2000–2010 in the United States after sustaining a TBI indicates that females had a 33.1% lower probability of death than males, after adjustment for multiple covariates; the adjusted probability of death for both sexes has been shown to increase with age up to ages 65–69 [29]. Based on analysis of data from a national trauma databank, TBI was more prevalent in men and was associated with lower GCS scores and higher ED ISS scores on admission [30].

### 1.2. Objectives

Despite this remarkable convergence of multiple clinical measures, there is little empirical evidence and no comprehensive predictive models for the prognosis of TBI patients. Based on the previous studies reviewed above, we hypothesized that a model we created that exploits machine learning can accurately predict the mortality of TBI patients from certain clinical measures (GCS, ISS, BP, HR, and PP) and demographics (age and gender).

## 2. Materials and Methods

### 2.1. Study Materials

The study cohort consisted of patients who visited the emergency department (ED) at a high-level first aid duty hospital in northern Taiwan from 1 January 2008 to 31 June 2018. The retrospective dataset was a subset of data from electronic health records (EHRs) in a trauma registry system at the above hospital, which registers more than 120,000 emergency visits per year. Personal information was removed from the data prior to analysis and was not included in reports for publication. The inclusion criteria were that the patient had to be at least 16 years old and diagnosed in the ED as having TBI according to the 9th edition of the *International Classification of Diseases*, *Clinical Modification* [ICD-9-CM], specifically sections 800.00–801.9 (fracture of the vault or base of the skull), 803.00–804.9 (other and unqualified and multiple fractures of the skull), 850.00–850.90 (concussion, loss of conscious), 851.00–851.90 (other unspecified head injury), and 852.00–853.10 (any intracranial hemorrhage following injury); the one exception was section 854 (intracranial injury of other and unspecified nature), omitted so we could rule out nontraumatic brain injury [4]. Patients less than 16 years old were excluded because of less mature brain development and because clinical treatments for this group, such as advanced respiratory airway management, could influence the research outcome [31].

### 2.2. Clinical Measures and Demographics

To predict the in-hospital mortality of TBI patients from demographics and physiological signals at ED admission, the candidate predictors were extracted from the electronic health record (EHR) dataset; the datapoints were mainly demographic characteristics and clinical measures. The variables in the registry system are gender, age, GCS, ISS, and vital signs (all on arrival at the ED) and in-hospital mortality. Together these measures constitute the variable we expected to be a significant predictor variable of mortality in our sample of traumatic brain injury patients. The rationale for this expectation is described in the background section.

### 2.3. Machine Learning Algorithms

Following the guidelines for the Scikit Learn Algorithm [32], we used the following classification algorithms to determine the best predictor of in-hospital mortality of TBI patients in the ED in our study: linear SVC, KNeighbors, naïve Bayes, and SVC Ensemble. The predictive model was built from the derivation of the cohorts in two steps. First, several advanced linear or nonlinear machine learning algorithms were adopted to construct the following models: J48, random forest (RF), random tree (RT), reduce error pruning tree (REP Tree), k-nearest neighbors (KNN), naïve Bayes (NB) and support vector machine (SVM). J48, one of the most useful decision tree classification approaches, classifies patients using a divide-and-conquer strategy for decision making [33]. RF is an ensemble machine learning method that involves construction of multiple criteria via bootstrap aggregation that is well suited to perform embedded feature selection [34]. RT is an algorithm for constructing a tree that uses information obtained by splitting criteria but performs no pruning procedures [35]. REP tree is a fast decision-tree learner that builds a decision/regression tree using information obtained from the splitting criterion, and prunes it using the reduced error method [36]. Nearest neighbor classifiers are based on learning by analogy: when given an unknown sample, a k-nearest neighbor (KNN) classifier searches the pattern space for the k training samples that are closest to the unknown sample [37]. The NB classifier assumes that each variable is unrelated to the presence of any other variable; because the independent variables are assumed, only the variances of the variables for each class need to be determined [37]. SVM produces a model that predicts the target data value using a testing set that contains only attributes; it can classify both linear and nonlinear data [37].

### 2.4. Statistical Analyses and Machine Learning Framework

Results on the physiological signals and demographic characteristics (SBP, DBP, HR, PP, GCS, ISS, gender, age) were summarized as frequencies, percentages, means, and standard deviations. Independent t tests were used to analyze the differences among the demographics and physiological signals in predicting in-hospital mortality. Machine learning-based algorithms were used to express a sequential classification and are described by a set of attributes. The machine learning classification algorithms (J48, RF, RT REP Tree, KNN, NB, and SVM) were then evaluated. The algorithms were tested using Weka 3.8.3 open-source data mining software (Hamilton, New Zealand) [33].

The samples were randomly divided into 10 subgroups, 9 of which involved training and the other testing. The nine training subgroups were then combined, leaving one training and one testing cohort. The training and testing datasets were constructed using a pure random 10-fold cross-validation [38]. The following statistical classification measures were used for the evaluations: precision, recall, F1 score, true positive rate (TPR), false positive rate (FPR), area under the receiver operating characteristic curve (AUC), accuracy [33], and average success rate of classification [36].

### 2.5. Ethical Considerations

In agreement with the ethical requirements, it was emphasized to the participants’ that their data were confidential and would be used only for the purpose of this study. The hospital’s ethics committee approved the study (Approval No. 2-108-05-005).

## 3. Results

### 3.1. Sample Characteristics

**Figure 1:**
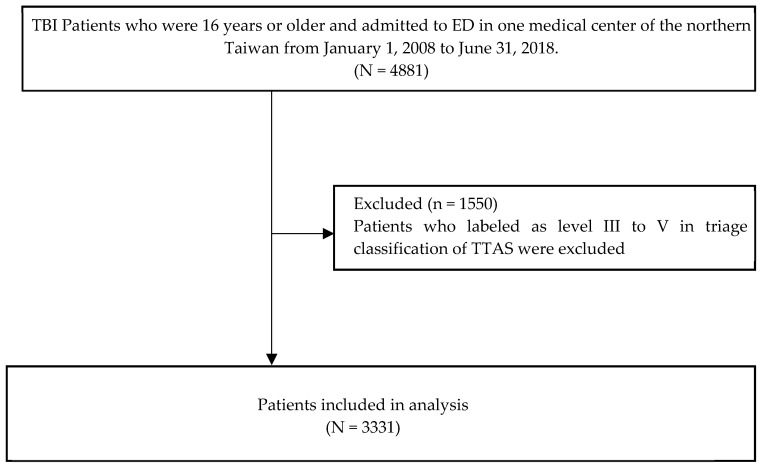
Flowchart of the patient selection process.

Of the 4881 TBI patients who were screened at the ED, 3331 were admitted and assigned to Level I, “resuscitation” (immediate) or Level II “emergent” (within 10 min) in triage, using the five-level Taiwan triage and acuity scale (TTAS) [39]. These 3331 patients became the sample for the study (Figure 1). There were no missing data.

The sample characteristics are listed in Table 1. Most participants were men (*n* = 2221, 66.7%) and the overall mean age was 51.14 years (SD = 29.17). Most survived (*n* = 3003, 90.15%) and the survivors had significantly higher SBP/DBP, PP, HR, GCS, and ISS and were significantly younger than those who died.

### 3.2. Performance of Classification Algorithms

Several machine learning-based algorithms were used in this research. The algorithm that achieved the best identification performance, based on its accuracy, precision, recall, F1 score, and average success rate of classification, was chosen as the best algorithm for modeling prediction of the mortality rate for TBI patients. The chosen algorithm was J48, its classification expressed as a tree (Table 2). It has an average success rate of classification of 77.2%, an accuracy rate of 93.2%, and an F1 score of 92.9%. According to the confusion matrix for the J48 algorithm (Table 3), the prediction algorithm we developed placed 188 of the 276 TBI patients who died (68.1%) in the correct category. It placed 2915 of the 3055 patients who survived (95.4%) in the correct category.

### 3.3. Algorithms for Predicting the In-Hospital Mortality of TBI Patients

**Figure 2:**
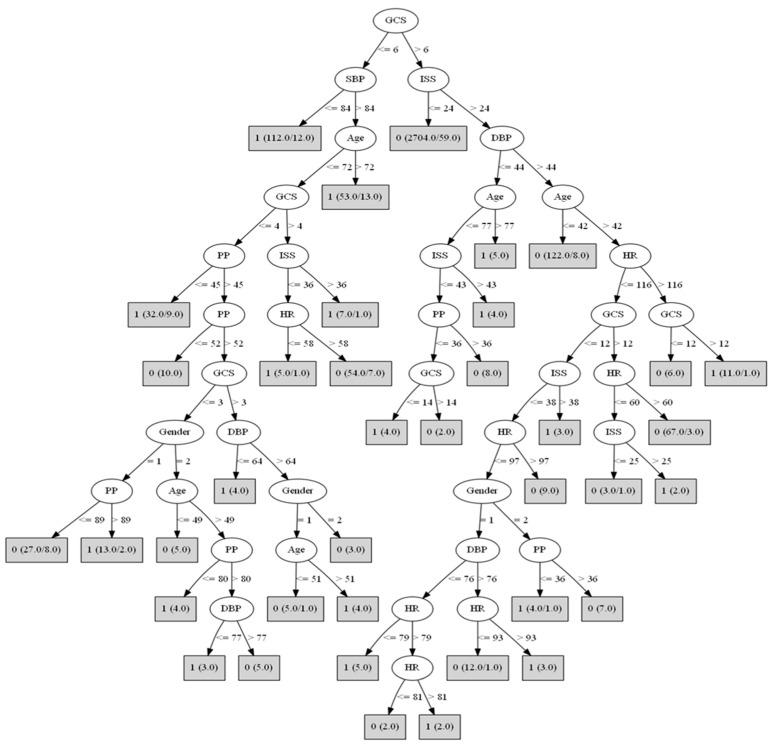
J48 Decision tree for predicting the in-hospital mortality of TBI patients. The root of the tree starts at the first variable (GCS). Since GCS is ≤6, the next variable in the tree is SBP and so on. Because SBP was ≤84, the patient was predicted to die, as indicated by “1” inside the gray box. The (112/12) means that 112 patients in the training set in this path were correctly classified (outcome = 1) and 12 were incorrectly classified (outcome = 0). GCS: Glasgow coma scale. ISS: injury severity scale. DBP: diastolic blood pressure. SBP: systolic blood pressure. PP: pulse pressure difference. HR: heart rate. Gender: 1 = male, 2 = female. Predicted outcome: 1 = died, 0 = survived.

The structure of the predictive algorithm we created is shown in Figure 2. The first splitting node is occupied by GCS scores, and the next splitting node by the SBP and ISS. TBI patients for whom GCS ≤ 6 and SBP ≤ 84 mm Hg were predicted to die (coded as 1 in the figure). In the first gray box in the figure, 1 is the prediction; (112/12) means that 112 patients from the training set in this path were correctly classified by the rule corresponding to the leaf, and 12 refers to the number of such patients misclassified by the rule. TBI patients for whom GCS > 6 and ISS ≤ 24 were predicted to survive (coded as 0 in the figure). Therefore, the best predictor variable is consciousness level as assessed by GCS, the second best are ISS and SBP, followed in order by age, DBP, PP, HR, and gender.

## 4. Discussion

### 4.1. Summary of the Results

Results from the study show that it is possible to predict the in-hospital mortality in TBI cases from demographics and clinical measures at ED admission using a novel machine learning-based algorithm. The prognostic model for TBI presented above is based on clinical traumatic registry information and physiomarkers in the ED. Given that our aim was to create a valid algorithmic model that can be applied successfully in the ED, we chose for our algorithm eight main variables that seemed to fulfill our aim. The chosen clinical measures (GCS, ISS, vital signs) overcame the major practical challenges presented by using complicated scoring tools in a clinical setting. The algorithm works even in conditions when monitoring is intermittently disrupted, as happens often in the ED. In the present study, the J48 algorithm’s performance and its average success rate of classification was good. The algorithm represents a promising start in the development of large-data algorithms for predicting TBI prognosis in the ED. Such algorithms have the potential to help clinical professionals and family members make timely, data-driven, and valid treatment decisions.

### 4.2. The Machine Learning Algorithms

The best way to assess the performance of prognostic models has not been conclusively determined. Traditionally, the performance of a prognostic model is tested by assessing its discrimination tools (i.e., AUC for binary outcome models) and calibration (i.e., probability estimate of a data point belonging to a class) [40]. The performance metrics include both threshold metrics (accuracy, F1 score, Cohen’s kappa), and rank metrics (AUC) [41]. One big difference between the AUC and F1 score is that the AUC predicts scores and the F1 score predicts membership in a class. However, for TBI prognosis algorithms, the changes in the predictions need to be properly calibrated probabilities and the dataset is heavily unbalanced. Thus, an AUC assessment could be misleading [41]. Therefore, the F1 score was chosen for this study so as to optimize the model’s performance.

Moreover, AUC has limited clinical interpretability since it does not account for the various misclassification costs arising from false-negative and false-positive diagnoses [42]. For TBI prognosis algorithms, successful diagnosis for the class “died” is the most important metric for clinicians trying to decide which emergent treatment to immediately offer TBI patients to prevent death. Therefore, the average success rate of classification was determined based on the demonstrated sensitivity and specificity of clinical consequences in predicting TBI mortality. It is noteworthy that the J48 algorithm had a maximum AUC > 80%. This shows that the use of prognostic algorithms does not mean that we should abandon AUC just because it is intended for a different purpose.

Regarding the prognosis model’s calibration, the algorithm that produced the highest accuracy was chosen as the most successful algorithm for modeling the prognosis of TBI patients. The most accurate classification rate overall (93.3%) was produced using the random forest decision algorithm, but the tree was not as successful in classifying both surviving and dying patients. We found that the random forest algorithm underestimated the risk of in-hospital mortality for those with a very low probability of death. Such underestimation (i.e., false negative) should always be interpreted with caution, as a poor prognostic estimate can easily become a self-fulfilling prophecy for surviving TBI patients. Therefore, the average success rate of classification was calculated for the two models that gave the best average success rate of classification (J48 and Random Forest), and the higher average rate was found to be achieved by the model relying on the J48 algorithm (see [36]).

The benefits of predicting mortality in TBI patients from GCS scores have been questioned [10]. The present algorithms included GCS scores as one of their most important components. Of the eight clinical variables in the algorithms, three were blood pressure-derived measures, and two were injury-derived. GCS was the top-most variable in the tree (accounting for the highest incidence of TBI mortality), highlighting the importance of the cutoff value of 6 for GCS. However, the current standard definition of a severe TBI patient (GCS ≤ 8) has the potential of placing TBI patients with GCS ≤ 6 and GCS 7–8 at the same risk of mortality, as the currently accepted trauma triage relies on abnormal level of consciousness criteria such as GCS < 8 to determine a patient’s advanced airway mode, priority of treatment, destination for treatment, and need for possible life-saving interventions [9]. Thus, based on the current guideline of GCS ≤ 8 for severe TBI patients, we should consider such patients for advanced medical resuscitation. Moreover, we should pay more attention to TBI patients with GCS ≤ 6, because the optimal prediction threshold from our data-driven best fit algorithm suggests a high incidence of in-hospital mortality for such patients.

The SBP variable in the algorithm was defined solely by professional assessment and data monitoring, leaving no room for subjective interpretation of the patient’s clinical condition. In previous studies, hypotension was defined as SBP < 90 mm Hg or <110 mm Hg [24]. Our results indicate that SBP ≤ 84 mm Hg is a better predictor of TBI prognosis, as seemingly are hemodynamic features and trends measured later. Such hemodynamic features are easy for a clinician to measure, and they take into account, on a daily basis, resuscitation in triage or treatment in the ED; the values are even comprehensible when computed using a machine learning approach. Hemodynamic status, as inferred from measures such as SBP, is represented in our algorithm by a single binary variable of hypotension. According to our results, patients with GCS ≤ 6 should be considered high risk for in-hospital mortality if SBP < 84 mm Hg. Worth mentioning also is that the four hemodynamic variables in the J48 algorithm (SBP, DBP, SS and HR) were defined by the results of data monitoring, highlighting the importance of objective clinical assessment.

Although the results of the present study seem to be no different than the results of other studies that predicted TBI mortality from GCS, ISS and vital signs, none of these studies provided valid cutoff values for all these important clinical measures at ED admission. One study did provide a cutoff value for the ISS [13], and it was similar to the cutoff value we obtained from the J48 algorithm. ISS scores can range from 1–75, with a higher value indicating a more severe injury. In our study, TBI patients with ISS > 24 were predicted to have a higher incidence of in-hospital mortality, based on their classification scores for severity of injury in all regions (head, face, neck, thorax, abdomen, spine, upper and lower extremities, and external/other) as measured after the initial examination. Except for SBP and GCS, it can be argued that prognostic variables are less meaningful if measured at admission than at triage or clinical nursing assessment, although it can be argued that the addition of ISS to SBP and GCS increases the algorithm’s discrimination. However, ISS has been shown to be the more important predictor for shared decision making with respect to treatment and resuscitation [5]. The prognostic importance of ISS is further highlighted by the appearance, several times, of the values 36, 38 and 43 in the algorithm, because they were derived from data; we can develop these algorithms further through multinational collaboration. As mentioned before, it is important to note that the categories of TBI severity have a huge impact on the mortality numbers. Our result that ISS > 24 predicts a higher risk of in-hospital mortality is helpful in verifying the accuracy of the scoring tools.

It is important to note that two of the demographics, gender, and age, were important predictors of TBI mortality, a finding in line with a previous study [28]. Our results show that the highest incidences of in-hospital mortality were among older patients (i.e., age > 72 years with GCS ≤ 6 and SBP > 84 mm Hg; and age > 77 years with GCS > 6, ISS > 24, and DBP ≤ 44 mm Hg). Overall, older males had a higher mortality rate than older females. That age was a significant predictor suggests that more information about patients’ intracranial physiology makes it substantially easier to discriminate between survivors and non-survivors. In short, it is likely that increased incidence of in-hospital mortality is associated with being male and having the poor hemodynamic status that accompanies old age. One previous study revealed a higher probability of death in male TBI patients [29], but another study found no gender difference in the prediction of in-hospital mortality with a sample limited to severe TBI patients [28]. Haring et al. [29] tested TBI patients age ≥ 65 years but did not test severe TBI patients, whereas Areas et al. [28] tested TBI patients with GCS ≤ 8, which means their TBI was severe. It seems that the difference in the two samples with regard to TBI severity explains the high prevalence of mortality in male patients in Haring et al. [29]. Our results suggest that patients who have poor hemodynamic status or are elderly have the highest incidence of death. It is consistent with our study results that the effect of gender on the prediction of TBI mortality is in the branch nodes of the tree.

### 4.3. Implications of the Findings

These results provide health professionals with important information that should encourage them to pay more attention to the sequencing of physiological signals and clinical measures at ED admission in TBI patients so as to prevent death in these patients. Prognostic predictions based on data-driven algorithms could be used to alert emergency room nurses about subtle conscious and hemodynamic changes and the need to quantify the effects of different demographics for prognoses. It is important to note that GCS ≤ 6, and SBP ≤ 84 mm Hg are the reference values that should be used for decision-making in triage assessment and for emergency resuscitation. Including ISS > 24, which indicates an extreme, abnormal injury, would make it substantially easier to discriminate between survivors and non-survivors.

It has been shown that machine learning methods can usually be used by clinical professionals to predict patient outcomes and prognoses. The data-driven algorithms can be applied as components of bedside monitoring systems to assist clinicians with auto-alarm mechanisms and with immediate precision-care. Moreover, it is possible to distinguish high mortality TBI patients at ED triage and to determine optimal courses of treatment and optimize patient outcomes. Because we used precise decision-tree algorithms, the results of our study have important implications for clinical education by aiding physicians and nurses in making triage decisions that are more data-driven and improving shared decision making with TBI patients and their families, which in turn may result in better quality of care.

### 4.4. Limitations

Although these findings are noteworthy, several limitations should be taken into account. First, although a machine-learning prognostic model demonstrated the most reliable and consistent results, the fact that the predictors all came only from patients admitted to an ED may have affected the generalizability of the findings, since patients could have undergone different treatments following our treatment. It is possible the model would work better if subsequent treatment and clinical manifestations in advanced treatment contexts are taken into account. Moreover, most of the empirical research on TBI mortality has been retrospective; future research should examine prospectively the prediction performance of our algorithms. In addition, the results of this study are exploratory, and the study was conducted at a single site, which limits the generalizability of our findings. In addition, the study was limited by the small number of patients who died, meaning that their proportion of the total sample is not a reliable estimate of the population value. Additionally, because of the retrospective nature of the study and the limitations of the database, the model did not take into account other potentially influential variables such as underlying medical conditions, comorbidities, type of treatment received in the ED or in hospital, and imaging findings. We suggest further prospective studies to solve this problem. Finally, because of the relatively long-term time frame in this retrospective study, some potentially confounding factors such as use of new techniques or treatments, type of provider, and changes in the protocol, treatment guidelines or other treatment strategies, we did not examine whether the above interventions were performed. Thus, we could not properly assess the impact of these confounding factors on the clinical outcomes. We nevertheless believe that our approach is advantageous in the early stages of developing new clinical algorithms, as it gives us a better understanding of how such algorithms work and which variables are truly valuable for outcome prediction.

## 5. Conclusions

The results from our algorithm for predicting mortality of patients with TBI treated in the ED provide clinical professionals with effective cutoff values for the relevant clinical measures at ED admission. The simple algorithms are based on eight main variables and, in contrast to current prognostic criteria, provide evidence from data-driven machine learning and may aid in clinical decision making. These results provide healthcare professionals with important information to increase their awareness of the sequences of clinical prognosis in TBI patients, preventing possible death. Highly accurate classification rates of prognosis prediction for TBI patients that use decision tree algorithms guide clinical health professionals in assessing and offering intensive care and in shared decision making with family members of TBI patients.

## Figures and Tables

**Table 1:** Sample characteristics and differences in mortality of patients ^a,b^.

Variables	All (*N* = 3331)		Lived (*n* = 3003)		Died (*n* = 328)		*p*-Value ^c^
	M	SD	M	SD	M	SD	
Gender (*n*, %)							0.622
Male ^a^	2221	66.68	1998	90.00	223	10.00	
Female ^a^	1110	33.32	1005	90.54	105	9.46	
Age	51.14	29.17	50.42	29.66	57.79	23.24	<0.001
SBP	137.54	42.03	140.64	33.42	109.15	82.74	<0.001
DBP	78.70	23.93	80.79	19.83	59.52	42.60	<0.001
PP	58.84	27.53	59.85	22.80	49.63	53.38	<0.001
HR	85.00	24.09	86.27	19.85	73.37	46.30	<0.001
ISS	16.09	8.64	14.95	7.38	26.53	11.78	<0.001
GCS	12.97	3.70	13.69	2.78	6.36	4.44	<0.001

^a^ Values are *n* under M and % under SD. ^b^ SBP: systolic blood pressure. DBP: diastolic blood pressure. PP: pulse pressure difference. HR: heart rate. ISS: injury severity scale. GCS: Glasgow coma scale. M: mean. SD: standard deviation. ^c^ *p*-values are for the differences in sample characteristics between the lived and died groups from Chi-square (gender) and independent *t* tests.

**Table 2:** Calculated results of average success rate of classification (%).

Model	Accuracy	Precision	Recall	F1	AUC	Success Rate, Class “Lived”	Success Rate, Class “Died”	Average Success Rate
J48	93.2	92.7	93.2	92.9	82.0	97.1	57.3	77.2
Random Forest	93.3	92.7	93.3	92.9	92.1	97.8	52.4	75.1
Random Tree	91.0	90.8	91.0	90.9	73.5	95.2	51.8	73.5
REP Tree	92.0	91.1	92.0	91.4	84.6	96.9	46.3	71.6
KNN	91.0	90.5	91.0	90.7	71.6	95.7	48.2	72.0
SVM	93.2	92.5	93.2	92.3	71.0	98.7	43.3	71.0
NB	91.9	91.7	91.9	91.8	91.7	95.7	56.4	76.1

AUC: area under the receiver operating characteristic curve. REP tree: reduce error pruning. KNN: k-nearest neighbor. SVM: support vector machine. NB: naïve Bayes.

**Table 3:** Confusion matrix of J48 algorithms.

Outcomes		Predicted Outcome	
		Alive	Died
Actual outcome	Alive	2915	88
	Died	140	188

## Data Availability

Not applicable.
